# German Transcreation of the International Standards to Document Remaining Autonomic Function After Spinal Cord Injury (Second Edition): A Feasibility Study in Individuals With Subacute Phase Spinal Cord Injury/Disease (SCI/D)

**DOI:** 10.46292/sci25-00010

**Published:** 2025-08-22

**Authors:** Carole Niederberger, Elena Henes, Andrei V. Krassioukov, Michael Baumberger, Jörg Krebs, Jürgen Pannek, Matthias Walter, Anke Scheel-Sailer

**Affiliations:** 1 Swiss Paraplegic Centre, Nottwil, Switzerland; 2 International Collaboration on Repair Discoveries, University of British Columbia, Vancouver, Canada; 3 Department of Urology, University Hospital Berne, University of Berne, Switzerland; 4 Department of NeuroUrology, Swiss Paraplegic Centre, Nottwil, Switzerland; 5 University of Basel, Faculty of Medicine, Basel, Switzerland; 6 Swiss Paraplegic Research, Nottwil, Switzerland; 7 Berne Rehabilitation and Sport Department, University Hospital Berne, Switzerland

**Keywords:** autonomic nervous system disease, feasibility studies, humans, nervous system disease, neurological examination

## Abstract

**Background:**

In May 2021, the second edition of International Standards to document remaining Autonomic Function after Spinal Cord Injury (ISAFSCI) was published.

**Objectives:**

To transcreate the 2021 ISAFSCI (2nd ed.) to German and to assess its feasibility in the subacute phase following spinal cord injury/disease (SCI/D).

**Methods:**

Transcreation to German was performed by an interdisciplinary team of native English and German speakers. We screened individuals with SCI/D for eligibility (i.e., age ≥18 years; >3 months following SCI/D) between August 2021 and January 2022. To minimize the time for the assessment, we first interviewed participants in a supine position before conducting the clinical examination. We assessed participants thrice within 14 days using a randomized sequence of assessors, and we assessed SCI/D according to the International Standards for Classification of Spinal Cord Injury (ISNCSCI) grading of the SCI/D, including the American Spinal Injury Association Impairment Scale (AIS). Time of ISAFSCI assessments (median and quartiles) and its completeness (%) were calculated.

**Results:**

Twelve participants (3 females; median age 42 years [Q1: 31, Q3: 54]) were enrolled and assessed thrice. Severity and level of injury were either sensorimotor complete (AIS A, 9) or incomplete (AIS C, 3) SCI/D and tetraplegia (*n* = 5) or paraplegia *(n* = 7), respectively. Median time to complete an assessment was 39 minutes (Q1: 32, Q3: 46).

**Conclusion:**

The German version of the ISAFSCI second edition is feasible to perform in a subacute cohort. However, given the subacute stage following SCI, certain limitations must be acknowledged. Many participants have not yet engaged in sexual activity, which limits the evaluation of sexual function.

## Introduction

Spinal cord injury (SCI) is characterized by motor, sensory, and autonomic impairments depending on the magnitude of the damage to the spinal cord.^1^ Understanding the consequences of injury to these respective pathways is an integral part of establishing therapy and rehabilitation goals for individuals with SCI.^2^ In clinical practice, the International Standards for Neurologic Classification of SCI (ISNCSCI) are already a well-established assessment tool.^3^ However, the ISNCSCI does not assess the autonomic nervous system (ANS).^2^ The ANS dysfunction after SCI presents a significant challenge both clinically and in terms of quality of life for individuals with SCI.^4^–^7^ Furthermore, ANS dysfunction underpins many of the medical complications seen in individuals with SCI.^8^–^10^

In 2021, the second edition of International Standards to document remaining Autonomic Function after SCI (ISAFSCI) was published, introducing new standardized assessments of various components of the ANS. Blood pressure, heart rate, body temperature, and respiration function are objectively measured and can be self-assessed by an individual with SCI.^11^ While feasibility and reliability of ISAFSCI (first edition) in the chronic SCI population (>1 year post injury)^12^,^13^ has been demonstrated, there is no evidence-based recommendation regarding the routine implementation of ISAFSCI during the acute and subacute phases after SCI. Thus, a reliable method for assessing and documenting residual autonomic function after SCI is still missing.^12^–^14^

Considering the interrater reliability of the first ISAFSCI,^12^ the initial rehabilitation guidelines of the German-speaking society for SCI (DMGP) encouraged the use of the first ISAFSCI version across all SCI centers in Germany, Switzerland, and Austria. Following the publication of the second edition, the DMGP advised all associated SCI centers to continue using the first edition until further notice. To incorporate the documentation of autonomic function as a standard component of the neurological assessment during initial rehabilitation, and for implementation in DMGP-associated SCI centers, the second edition of ISAFSCI is needed in German for use in the acute and subacute phases following SCI.^12^–^15^

Thus, the primary objectives of this study were to create an adapted German version of the ISAFSCI, second edition, and to evaluate its feasibility in the subacute phase following SCI, specifically within an inpatient setting. We hypothesized that it would take less than 60 minutes, including clinical examination and questionnaire, and that the completeness of all questionnaire items would be ≥90%. Further, a single person should be able to perform the assessment alone, without additional help.

## Methods

### Study design and ethics

This study was approved by the Ethics Committee of Northwest and Central Switzerland (EKNZ ID 21-01284). We followed the STARD checklist (https://www.equator-network.org/reporting-guidelines/stard/) to ensure comprehensive and transparent reporting.

### Objective, outcome variables, and samples size

Our objectives were to transcreate the second edition of ISAFSCI to German and to assess its feasibility in the subacute phase following SCI/disease (SCI/D).^11^ We collected sociodemographic data (e.g., age, sex, and education), injury characteristics (e.g., severity and neurological level of injury in accordance with the ISNCSCI grading the SCI/D, including the American Spinal Injury Association Impairment Scale [AIS]), ISAFSCI scores, and overall time of conducting an ISAFSCI assessment. Our target number of participants (*N* = 12) for this feasibility study was a convenience sample and in line with reports from the literature on feasibility in individuals with SCI.^16^–^19^

### Participants and study location

**Figure 1.**
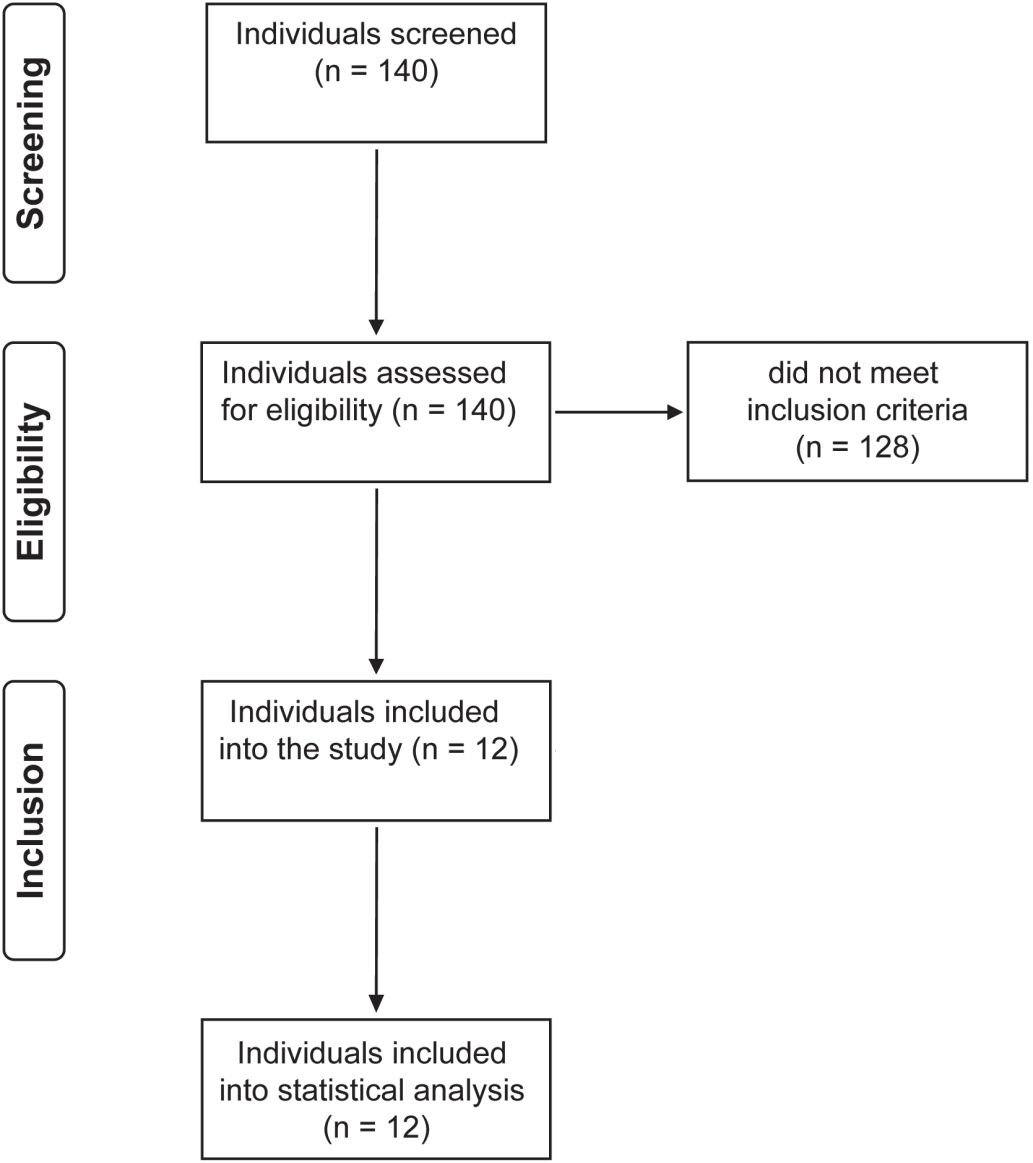
Study flow diagram.

Individuals were screened for eligibility between August 4, 2021, and January 31, 2022 (**Figure 1**). Inclusion criteria were age ≥18 years with no upper limit, subacute phase (i.e., >3 months) after SCI/D and >2 weeks before discharge from the initial rehabilitation, willingness and ability to comply with all study-related assessments, ability to understand and complete study-related questionnaires (able to understand and speak German or have access to an appropriate interpreter as judged by the investigator), and signed informed consent. Exclusion criteria were acute infection or health condition that, in the investigator's judgment, would adversely affect the participant, (progressive) neuroinflammatory disease (e.g., Guillain-Barré syndrome, critical illness, polyneuropathy, multiple sclerosis), relevant communication impairment, and having any relevant cognitive impairment (Montreal Cognitive Assessment [MoCA] < 26). The latter MoCA cutoff was in line with the original MoCA validation study to define normal cognitive performance.^20^

### Transcreation process

Prior to this study, an in-depth transcreation of the original version of the ISAFSCI^2^ was performed in accordance with Epstein et al.^21^ as well as Sousa and Rojjanasrirat.^22^ Our interdisciplinary team of native English and German speakers included representatives from various fields and specializations who, in our view, had relevant expertise, such as urologists, physiatrists, neurologists, researchers, and nurses. We utilized a forward and backward translation, revisions, and finalization process as outlined by Epstein et al.^21^ and Sousa and Rojjanasrirat.^22^ Thus, the transcreation process of the ISAFSCI second edition,^11^ including its tables and questions, was adapted accordingly. The comparison with the first edition of ISAFSCI also fed into our transcreation process (see Supplementary Methods, eTable 1). The transcreated content, including the questionnaire, underwent several test runs prior to its implementation in the current feasibility study (see Supplementary Methods).

### Adaptation of the assessment

The aim was to evaluate participants (i.e., inpatients) during the subacute phase after SCI/D in accordance with the recommendations from the second edition of ISAFSCI. To facilitate the completion of the assessment within 60 minutes, we developed a worksheet that enables a single individual to carry out all tasks efficiently. After providing written informed consent, enrolled participants were scheduled to undergo 3 assessments within 14 days. Two assessors conducted the assessments in a randomized fashion (twice by assessor 1, once by assessor 2) to limit the risk of sequence bias. All assessments and interviews were conducted at our SCI/D-specialized acute care and rehabilitation center. Prior to the assessment, assessors conducted several test runs to optimize the time needed to perform the assessment. Thus, we first interviewed participants in a supine position before conducting the clinical examination. The neurological examination was shortened to the essential dermatomes determining the ISAFSCI scores. Each assessment was performed by the assessor alone without additional assistance. The ISAFSCI score sheet was completed during the assessment. Thereafter, coded data were stored in our data management system (secuTrial; iAS, Berlin, Germany).

### Assessment preparation and questionnaire

The date and the starting and end times of the assessment (questionnaire and measurements) were documented. At the start of each assessment, the participant was in a supine position in bed, wearing a maximum of one layer of indoor garments. A blood pressure cuff was placed on the left upper arm, and 3 ECG electrodes were placed on the upper body (Nellcor N5600 multiparameter monitor; Medtronic, USA). Any protocol deviation was noted. At the beginning of every assessment, we asked the participants if any new health problems had appeared and if the participant was ill or had a fever. The following information was recorded: room temperature, intake of caffeine, large meals, heavy training, nicotine, cannabis, and alcohol during the last 4 hours before the assessment, last time of bladder emptying and bowel movement, and wearing of compression stockings or an abdominal belt. Further details on the actual assessment can be found in the Supplementary Methods.

While the participant remained in a supine position, the assessor continued with the assessment part of the questionnaire (see Supplementary Methods, eTable 2). The participant was free to respond openly, while the assessor selected the most appropriate answer.

### ISAFSCI score sheet

The assessor completed the ISAFSCI score sheet only after gathering information from the assessment and questionnaire. Additional information was required for the bronchopulmonary section. Subsection scores were obtained for the general autonomic function part (i.e., cardiovascular, thermoregulation, sudomotor, and bronchopulmonary system). For the sacral autonomic function part, anticipated functional score (i.e., based on the neurological level of injury as obtained on the ISNCSCI) and participant-reported scores were determined.

### Roundtable meeting after assessment completion

After having completed all assessment of all participants, we held a roundtable meeting to discuss feedback from all assessors, addressing any peculiarities or issues that had arisen. This collaborative discussion aimed to adapt tasks and refine the transcreation process for future assessments.

### Data extraction and statistical analysis

Medical data were extracted from the hospital medical information systems (MedFolio version 2.2.0.2482; nexus ag, Switzerland). Data are presented as count and percentages. Nonparametric descriptive statistics were applied. Results are presented as median with lower (Q1) and upper (Q3) quartiles as well as minimum and maximum. Completeness of the second edition ISAFSCI scores (including the proportions for not tested [NT] or not stated) are presented in percentages. The assessors were blinded to each other's assessments (i.e., ISAFSCI scores) and time taken for the assessment. Results are presented in accordance with International Spinal Cord Injury Core Data Set (version 3.0).^23^

## Results

### Participants, sociodemographic, and injury characteristics

Twelve participants (3 female [25%], median age 42 years [Q1: 31, Q3: 54, 24-70]) were enrolled (**Figure 1**) and assessed thrice. Severity and level of injury were either sensorimotor complete (AIS A, 9) or incomplete (AIS C, 3) SCI/D and tetraplegia (*n* = 5) or paraplegia (*n* = 7). Eight participants had a traumatic SCI, while 4 had a nontraumatic SCD. The median time since onset of SCI/D was 126 days (Q1: 108, Q3: 157, 94-202) (**Table 1**).

**Table 1.** Participant characteristics and time per assessment

ID	Age, years	Sex	AIS	NLI	Time since SCI/D, days	Time per assessment, minutes			
						1st	2nd	3rd	Median
1	60-64	Male	A	C6	202	45	55	60	55
2	40-44	Female	A	T10	183	25	27	32	27
3	30-34	Male	C	L2	121	45	40	35	40
4	25-29	Male	A	C7	183	50	50	30	50
5	20-24	Male	A	C6	148	50	36	42	42
6	25-29	Male	A	T10	112	50	35	30	35
7	50-54	Female	C	T8	148	65	40	35	40
8	55-59	Male	A	T6	95	45	30	44	44
9	70-74	Male	C	C5	118	65	35	25	35
10	50-54	Male	A	C3	132	45	40	60	45
11	30-34	Male	A	T7	94	35	38	30	35
12	40-44	Female	A	L1	95	30	35	28	30
Median	42	_-_	_-_	_-_	126	45	37	34	_-_

*Note:* To protect the privacy of the participants, we chose to provide a 5-year range (in line with the International Spinal Cord Injury Core Data Set Version 3.0) rather than revealing the actual age. AIS = American Spinal Injury Association Impairment Scale; ID = identification; NLI = neurological level of injury; SCI/D = spinal cord injury/disease.

### Assessment duration and ISAFSCI scores

The median time for each assessment was 39 minutes (Q1: 32, Q3: 46, 25-65), with examination times decreasing from the first assessment (45 minutes) to the third (34 minutes). Participants with tetraplegia required more time for an assessment (median 50 minutes [Q1: 45, Q3: 50, 45-65]; median 40 minutes [Q1: 36, Q3: 50, 35-55]; median 42 minutes [Q1: 30, Q3: 60, 25-60]) compared to those with paraplegia (median 45 minutes [Q1: 32, Q3: 48, 25-65]; median 35 minutes [Q1: 32, Q3: 39, 27-40]; median 32 minutes [Q1: 30, Q3: 35, 28-44]). Across all 3 assessments, a median of 91% (Q1: 86, Q3: 92, 68-100) of all items from the second edition ISAFSCI were recorded (**Table 2**). The general autonomic function items (cardiovascular, thermoregulation, sudomotor, and bronchopulmonary systems) were recorded at a median of 100% (Q1: 100, Q3: 100, 94-100) with only one incomplete participant. The anticipated functions of sacral autonomic function (lower urinary tract, gastrointestinal tract, and genital and reproductive organs) were recorded at a median of 100% (Q1: 96, Q3: 100, 78-100) with only 3 incomplete participants. Patient-reported functions of sacral autonomic function were recorded at a median of 76% (NT 24%; Q1: 63, Q3: 89, 46-100) with only 2 complete participants (**Table 3**).

**Table 2.** Completeness of ISAFSCI score across the 3 assessments

ID	Completeness of ISAFSCI score, %			
	1st	2nd	3rd	Median
1	95	86	91	91
2	91	91	91	91
3	100	100	100	100
4	77	86	86	83
5	95	91	86	91
6	100	91	91	94
7	86	82	77	82
8	95	95	100	97
9	77	91	86	85
10	82	91	68	80
11	100	100	100	100
12	86	86	82	85
Median	93	91	88	91

*Note:* ID = identification; ISAFSCI = International Standards to document remaining Autonomic Function after Spinal Cord Injury.

**Table 3.** Completeness of ISAFSCI score, sacral autonomic function, anticipated functional score (i.e., based on the NLI as obtained on the ISNCSCI), and patient-reported score

ID	Completeness of ISAFSCI score, %		
	General autonomic function	Sacral autonomic function	
		Anticipated functional score	Patient-reported score
1	100	100	75
2	100	83	88
3	100	100	100
4	100	100	54
5	100	100	75
6	100	100	83
7	100	83	63
8	100	100	92
9	100	100	63
10	94	100	46
11	100	100	100
12	100	78	75

*Note:* ID = identification; ISAFSCI = International Standards to document Autonomic Function following Spinal Cord Injury; ISNCSCI = International Standards for Classification of Spinal Cord Injury; NLI = neurological level of injury.

Notably, in the subsections, the highest percentage of nontestable items was observed in the patient-reported function of genitalia and reproductive systems (**Table 4**). Among individual questions combined over the 3 assessments, most nontestable items were related to orgasm (NT 58%) and ejaculation (NT 69%) within the patient-reported scores (**Table 4**). 

**Table 4.** Number of not tested or not stated items regarding each autonomic function for each assessment

Assessments	Number of not tested (NT) or not stated items, n (%)							
	1st		2nd		3rd		Total	
General autonomic function								
Cardiovascular system								
Heart rate	0		0		0		0	
Systolic blood pressure	0		0		0		0	
Diastolic blood pressure	0		0		0		0	
Thermoregulatory system								
Core body temperature	1 (8%)		0		0		1 (3%)	
Sudomotor system								
Sweating	1 (8%)		0		0		1 (3%)	
Bronchopulmonary system								
Inspiration/Ventilation	0		0		0		0	
Sacral autonomic function								
	Anticipated	Patient reported	Anticipated	Patient reported	Anticipated	Patient reported	Anticipated	Patient reported
Lower urinary tract								
Awareness of bladder fullness	0	0	0	0	0	1 (8%)	0	1 (3%)
Ability to prevent bladder leakage	0	1 (8%)	0	1 (8%)	0	1 (8%)	0	3 (8%)
Gastrointestinal tract								
Awareness of bowel fullness	0	0	0	0	0	0	0	0
Ability to prevent bowel leakage	0	1 (8%)	0	1 (8%)	0	0	0	2 (6%)
Genitalia and reproductive function								
Psychogenic arousal	0	4 (33%)	0	5 (42%)	0	3 (25%)	0	12 (33%)
Reflex genital arousal	0	2 (17%)	0	0	0	3 (25%)	0	5 (14%)
Orgasm	0	6 (50%)	0	7 (58%)	1 (8%)	8 (76%)	1 (3%)	21 (58%)
Ejaculation	3 (25%)	8 (67%)	3 (25%)	8 (67%)	3 (25%)	9 (75%)	9 (25%)	25 (69%)

### Key points of roundtable meeting and additional observations

Questionnaire:

- Sudomotor function: If the first question is answered with “anhidrosis,” no further questions should be asked. Occasionally, we observed that patients were asked further questions and then contradicted their previous answers.
- In general, participants expressed difficulties in adequately answering the questions, as there was no opportunity to address these topics during the initial rehabilitation phase. This was particularly evident concerning sexual function and sudomotor function.

Measurements:

- Body temperature: In three assessments, the body temperature was measured 3 times, even though only 2 measurements were necessary.
- Spirometry: Bedside spirometry was found to be quite complex and time-consuming for a task that does not influence the score on the ISAFSCI sheet.

Scoring sheet and assessment:

- Pulse measurement: It was unclear how to process the measured data for scoring purposes, including whether to take average values or consider the range (min/max).
- Sacral autonomic function, anticipated function, and sexual function: Certain combinations of assessment results created ambiguous scoring.

Supplementary Results provides a detailed list with all points and recommended adaptations.

## Discussion

### Feasibility

The results of this feasibility study show that the transcreated German version of the second edition ISAFSCI can be conducted effectively within a subacute inpatient SCI/D population. The median assessment time was 39 minutes, well below the hypothesized 60 minutes. Additionally, the high overall completeness rate of 90% for the ISAFSCI items, with the general autonomic function items achieving 100%, demonstrates the tool's comprehensive applicability. Two other studies have already successfully applied the cardiovascular section of the ISAFSCI, one of them even in a bedside setting.^24^,^25^ Thus, our positive results support the previous reports.

### Assessment time and efficiency

The assessments were conducted efficiently, with the median time decreasing from 45 minutes in the first session to 34 minutes by the third. This reduction underscores the enhanced efficiency with repeated use, likely due to both assessors’ and participants’ growing familiarity with the tool. It is important to note that the assessment time did not account for preparation time for devices and participants. Additionally, performance bias may be a concern, as both participants and assessors underwent a learning curve while completing 3 assessments within a short timeframe, which may not be easily replicable in a clinical setting.

### Completeness of ISAFSCI scores

The study achieved a high completeness rate for ISAFSCI items, demonstrating that most questions and examinations are both understandable and executable. However, challenges emerged in assessing the patient-reported function of sacral autonomic function, particularly concerning sexual function. A relevant proportion of sexual function questions were marked as “not tested” (NT), in particular for orgasm (58%) and ejaculation (69%). This finding underscores the need for targeted strategies to enhance the assessment of sexual health.

The elevated NT rates for sexual function questions may be attributed to various factors, including response bias. Participants might underreport or misreport their experiences due to social desirability, embarrassment, or misunderstandings of the questions. Explicit feedback from participants revealed that many felt they lacked the opportunity to test these functions during initial rehabilitation, while older participants often expressed disinterest in sexual functions, complicating data collection. Discussing sexual health poses challenges not only within the SCI/D population but also in broader contexts, requiring sensitivity and experience. Implementing specific training could facilitate more empathetic and competent discussions on these sensitive topics.

Study training on sexual function^26^ indicates that rehabilitation workers can benefit from customized training provided by a dedicated team focused on sexual health care. Strategically collaborating within a multidisciplinary team framework—defining tasks, determining proactive and reactive roles, and establishing formal agreements—seems essential for effectively integrating sexual health into the comprehensive care of patients.

### Key points of roundtable meeting

During the roundtable meeting, we identified additional critical points that were not addressed in the quantitative survey. In addition to the previously mentioned challenges in assessing sexual function, we encountered difficulties in evaluating sudomotor function, including contradictory statements and insufficient time to gain adequate experience in this area. As noted in the second edition, techniques for quantifying sudomotor function are not a suitable substitute and provide no real alternatives.^11^ Other questionnaires, such as COMPASS-31 and SCOPAAUT, appear to address this topic even more superficially and offer little benefit in the subacute setting.^27^,^28^ Thus, further research to optimize the questionnaire for the subacute phase is warranted.

Measurement issues included unnecessary multiple body temperature readings and complex bedside spirometry, which did not influence ISAFSCI scoring. It's worth mentioning that study participants undergo standardized body plethysmography as part of their initial rehabilitation; if these values prove comparable, body plethysmography could potentially replace spirometry in the future.^29^ Additionally, clarification is needed regarding the evaluation of pulse measurements and certain scoring ambiguities in sacral autonomic function assessments. Beyond the aforementioned adaptations to the assessment, we believe that structured training could enhance performance, as demonstrated in the first edition of ISAFSCI and ISNCSCI.^30^,^31^

## Limitations and Future Research

Our feasibility study yielded promising results; however, several limitations and biases need to be addressed. Our sample size (*N* = 12) restricts the generalizability of our findings, and we plan to conduct a follow-up study with 30 participants to further validate these results. Ensuring both intraand interrater reliability in future studies will be essential for establishing the robustness of the ISAFSCI. Information bias may arise from potential errors in data collection from hospital systems, which could impact the results. Additionally, the risk of reporting bias has been previously highlighted, particularly concerning the patient-reported sections of the second edition.

Another critical consideration for future validation studies is that, for most assessments (see Supplementary Methods, eTable 2), at least one measurable influencing factor on the autonomic nervous system (e.g., caffeine intake, wearing compression stockings) was applied, while the time of day for each assessment was merely documented. These factors should be thoroughly documented and accounted for in the study design and interpretation of the results.

## Conclusion

The German version of the second edition of ISAFSCI is feasible for application in the subacute phase of SCI/D, provided that specific adaptations and training are implemented for assessors. This assessment tool is both time-efficient and comprehensive, enhancing the monitoring and management of autonomic functions. By addressing the challenges identified during the study and conducting further validation, the second edition of ISAFSCI can be refined for routine clinical use, ultimately improving patient care and outcomes within the SCI/D population. Given the detailed and multifaceted nature of the assessment, structured training for assessors would be beneficial in the future.

## Supplementary Material




