# Supplementary material for: German Transcreation of the International Standards to Document Remaining Autonomic Function After Spinal Cord Injury (Second Edition): A Feasibility Study in Individuals With Subacute Phase Spinal Cord Injury/Disease (SCI/D)
Source: Top Spinal Cord Inj Rehabil. 2025 Aug 22;31(3):89–100. doi: 10.46292/sci25-00010 (PMC12376144; doi:10.46292/sci25-00010)
Supplement: Supplementary file 1 [file i1945-5763-31-3-89_s01.pdf]

eTable 1. ISAFSCI first Edition, English to German Transcreation

Original English version (ISAFSCI, 2017)	German version (SPZ, 2020)	Questions/Comments
**General Autonomic Function**	**Allgemeine Autonome Funktion**	
**Subsection 1 'Autonomic control of the heart'**	**A "Autonome Kontrolle des Herzens"**	Are multiple answers possible, e.g. bradycardia and tachycardia?
At rest, do you have a heart rate lower than 60 beats per minute?<br><br>(if yes, check 'bradycardia')	Haben Sie einen Ruhepuls (Herzschlag in Ruhe) von weniger als 60 Schlägen pro Minute?<br><br>(Wenn ja, wählen Sie "Bradykardie")	Should pulse be measured lying supine or sitting?
At rest, do you have a heart rate greater than 100 beats per minute?<br><br>(if yes, check 'tachycardia')	Haben Sie einen Ruhepuls (Herzschlag in Ruhe) von mehr als 100 Schlägen pro Minute?<br><br>(Wenn ja, wählen Sie "Tachykardie")	Should pulse be measured lying supine or sitting?
Have you ever experienced, or been told that you have an arrhythmia, or irregular heart rate?<br><br>(If yes, check 'other dysrhythmias')	Haben Sie jemals erlebt, oder wurde Ihnen gesagt, dass Sie einen unregelmässigen Herzschlag oder eine Herzrhythmusstörung haben?<br><br>(Wenn ja, wählen Sie "andere Rhythmusstörungen")	In the answer key, this is termed "other dysrhythmias" – what kind of arrythmias would be included here?<br>Ever means since the injury or including<br>before the injury?
(If 'I don't know' then check 'unknown'),<br><br>(If unable to assess, then check 'Unable to assess') (If 'no' to all, then check 'normal)	(Wenn "ich weiss nicht", wählen Sie "unbekannt") (Wenn nicht in der Lage zu beurteilen, wählen Sie "nicht in der Lage zu beurteilen")<br><br>(Wenn "nein" bei allen, wählen Sie "normal")	

**Subsection 2 'Autonomic control of blood pressure'**	**B "Autonome Kontrolle des Blutdrucks"**	Are multiple answers possible?
At rest, do you have a systolic blood pressure lower than 90mmHg?<br><br>(If yes, check 'resting systolic blood pressure below 90mmHg')	Haben Sie in Ruhe einen systolischen Blutdruck tiefer als 90mmHg?<br><br>(Wenn ja, wählen Sie "systolischer Blutdruck unter 90mmHg")	Should blood pressure be measured lying supine or sitting?

This supplementary digital material accompanies
Top Spinal Cord Inj Rehabil 2025;31(3):89-100
© 2025 American Spinal Injury Association
www.asia-spinalinjury.org
doi: 10.46292/sci25-00010

In the last 6 months, have you experienced, or been told that you have a condition called 'Orthostatic Hypotension'?<br><br>(if yes, check 'orthostatic hypotension')	Haben Sie in den letzten 6 Monate eine "orthostatische Hypotonie" erlebt, oder wurde Ihnen gesagt, dass Sie eine "orthostatische Hypotonie" haben?<br><br>(wenn ja, wählen Sie "orthostatische Hypotonie")	
Have you ever experienced, or been told that you have a condition called 'Autonomic Dysreflexia'?<br><br>(if yes, check 'Autonomic Dysreflexia')	Haben Sie jemals erlebt, oder wurde Ihnen gesagt, dass Sie eine "autonome Dysreflexie" haben?<br><br>(wenn ja, wählen Sie "autonome Dysreflexie")	Would it be helpful to add a non-medical definition of autonomic dysreflexia to the question?
(If 'I don't know' then check 'unknown')<br>(If unable to assess, then check 'Unable to assess') (If 'no' to all, then check 'normal)	(Wenn "ich weiss nicht", wählen Sie "unbekannt") (Wenn nicht in der Lage zu beurteilen, wählen Sie "nicht in der Lage zu beurteilen")<br>(Wenn "nein" bei allen, wählen Sie "normal")	

**Subsection 3 'Autonomic control of sweating'**	**C "Autonome Kontrolle des Schwitzens"**	
Do you sweat the same amount and as often now, compared to before your injury?<br><br>(if yes, then check 'normal' and no need to ask #2, #3, or #4)	Schwitzen Sie genauso viel und genauso oft wie vor Ihrer Verletzung?<br><br>(wenn ja, wählen Sie "normal" und #2, #3, #4 müssen nicht gefragt werden)	
Compared to before your injury, do you sweat a greater amount above the level of your injury?<br><br>(if yes, check 'hyperhydrosis above lesion')	Im Vergleich zu vor Ihrer Verletzung, schwitzen Sie vermehrt im Bereich oberhalb Ihrer Verletzungshöhe?<br><br>(wenn ja, wählen Sie "Hyperhydrose oberhalb der Läsion")	
Compared to before your injury, do you sweat a greater amount below the level of your injury?<br><br>(if yes, check 'hyperhydrosis below lesion')	Im Vergleich zu vor Ihrer Verletzung, schwitzen Sie vermehrt im Bereich unterhalb Ihrer Verletzungshöhe?<br><br>(wenn ja, wählen Sie "Hyperhydrose unterhalb der Läsion")	

Compared to before your injury, do you sweat a lower amount below the level of your injury?<br><br>(if yes, check 'hypohydrosis below lesion')	Im Vergleich zu vor Ihrer Verletzung, schwitzen Sie weniger im Bereich unterhalb Ihrer Verletzungshöhe?<br><br>(wenn ja, wählen Sie "Hypohydrose unterhalb der Läsion")	
(If 'I don't know' then check 'unknown')<br>(If unable to assess, then check 'Unable to assess')	(Wenn "ich weiss nicht", wählen Sie "unbekannt") (Wenn nicht in der Lage zu beurteilen, wählen Sie "nicht in der Lage zu beurteilen")	

**Subsection 4 'Temperature Regulation'**	**D "Temperaturregulation"**	
Has your ability to control your own body temperature changed since your injury?<br><br>(if 'no', check 'normal' and no need to ask #2 or #3)	Hat sich Ihre Fähigkeit Ihre Körpertemperatur zu regulieren sich verändert seit Ihrer Verletzung?<br><br>(wenn nein, wählen Sie "normal" und #2 und #3 müssen nicht gefragt werden)	
Do you have a difficult time remaining cool in a hot environment?<br><br>(if yes, then check 'hyperthermia')	Haben Sie Schwierigkeiten in einer heissen Umgebung kühl zu bleiben?<br><br>(wenn ja, wählen Sie "Hyperthermie")	
Do you have a difficult time remaining warm in a cool environment?<br>(if yes, then check 'hypothermia')	Haben Sie Schwierigkeiten in einer kühlen Umgebung warm zu bleiben?<br><br>(wenn ja, wählen Sie "Hypothermie")	
(If 'I don't know' then check 'unknown')<br>(If unable to assess, then check 'Unable to assess')	(Wenn "ich weiss nicht", wählen Sie "unbekannt") (Wenn nicht in der Lage zu beurteilen, wählen Sie "nicht in der Lage zu beurteilen")	

**Subsection 5 'Autonomic and somatic control of bronchopulmonary system'**	**E "Autonome und somatische Kontrolle des bronchopulmonalen Systems"**	
Has your ability to control your breathing/coughing/respiration changed since your injury?<br><br>(if 'no change', check normal and no need to ask #2-4)	Hat sich die Fähigkeit, Ihr Atmen und Husten zu beeinflussen und zu kontrollieren seit Ihrer Verletzung verändert?<br><br>(wenn nein, wählen Sie "normal" und #2-4 müssen nicht gefragt werden)	Is there a difference here between the meaning of breathing and respiration? we chose to exclude respiration in the German transcreation
Are you unable to voluntarily breathe without ventilator support?<br><br>(if yes, check 'unable to breathe requiring full ventilator support')	Sind Sie auf ein Beatmungsgerät angewiesen?<br><br>(wenn ja, wählen Sie "Spontanatmung beeinträchtigt, benötigt komplette Beatmungsunterstützung")	
Do you have impaired voluntary breathing requiring ventilator support part of the time?<br><br>(if yes, check 'Impaired voluntary breathing requiring partial vent support')	Ist Ihre Atmung beeinträchtigt und brauchten Sie teilweise ein Beatmungsgerät?<br><br>(wenn ja, wählen Sie "Spontanatmung beeinträchtigt, benötigt teilweise Beatmungsunterstützung")	
Do you have impaired voluntary respiration, but do not require ventilator support at any time?<br><br>(if yes, 'Voluntary respiration impaired, does not require vent support')	Ist Ihre Atmung beeinträchtigt, aber Sie brauchten zu keiner Zeit ein Beatmungsgerät?<br><br>(wenn ja, wählen Sie "Spontanatmung beeinträchtigt, braucht keine Beatmungshilfe")	
(If 'I don't know' then check 'unknown')<br>(If unable to assess, then check 'Unable to assess') (If 'no' to all, then check 'normal)	(Wenn "ich weiss nicht", wählen Sie "unbekannt") (Wenn nicht in der Lage zu beurteilen, wählen Sie "nicht in der Lage zu beurteilen")	

**'Lower urinary tract, bowel and sexual function'**	**"Untere Harntrakt-, Darm- und Sexualfunktion"**	
**Bladder**<br>Do you have an awareness of the need to empty your bladder?<br>Do you have the ability to prevent leakage (continence)?<br>What is your usual method for emptying the bladder?	**Harnblase**<br>Nehmen Sie wahr, wenn Sie Ihre Harnblase entleeren müssen?<br>Haben Sie die Fähigkeit Urinverlust (Kontinenz) zu vermeiden?<br>Was ist Ihre übliche Methode, um die Harnblase zu entleeren?	
**Bowel**<br>Do you have the sensation of need for a bowel movement?<br>Do you have the ability to prevent stool leakage (continence)?<br>Do you have voluntary anal sphincter contraction?	**Darm**<br>Spüren Sie, wenn Sie Ihren Darm entleeren müssen?<br>Haben Sie die Fähigkeit Stuhlverlust (Kontinenz) zu vermeiden?<br>Können Sie willentlich den Schiessmuskel des Enddarms (Analsphinkter) zusammenziehen?	
**Sexual Function**<br>Are you able to experience genital arousal (erection/vaginal lubrication/wetness)<br>By thinking sexual thoughts? By reflex or touch?<br>Are you able to experience orgasm? Are you able to ejaculate? (M only)<br>Do you have the sensation of menses? (F only)	**Sexualfunktion**<br>Können Sie Erregungen im Genitalbereich erleben (Gliedversteifung/ vaginale Sekretion)<br>Durch sexuelle Fantasien?<br>Als Reflex oder durch Berührung? Erleben Sie einen Orgasmus?<br>Können Sie ejakulieren (Samenerguss)? (nur Männer)<br>Spüren Sie Ihre Menstruation? (nur Frauen)	
Analysis:<br>Normal function = 2<br>Reduced or altered neurologic function = 1 Complete loss of control = 0<br>Unable to assess due to preexisting or concomitant problems = NT	Analyse:<br>Normale Funktion = 2<br>Reduzierte oder veränderte neurologische Funktion = 1<br>Komplett fehlende Funktion = 0<br>Aufgrund von vorbestehenden oder begleitenden Problemen nicht beurteilbar = NT	

First version of consensus-based transcreation, May 6, 2020 Process of transcreation: two independent (criteria) consensus

Questions/Comments/ feedback raised during the transcreation discussion

General feedback or questions: Do you ask for the whole time since injury or for a certain period? Subsection: Would it be helpful to add a non-medical definition of orthostatic hypotension to the question? Is there a reason why for bladder the term ‘awareness’ is used and for bowel the term ‘sensation’ is used?

eTable 2. Comparison first and second Edition of Transcreation English to German of ISAFSCI Question list

**First version of consensus-based transcreation from English to German of ISAFSCI Question list**			
**Original English version (ISAFSCI, 2017)**	**German version (SPZ, 2020)**	**ISAFSCI Version 2021, English**	**ISAFSCI Version, German, SPZ 2021**
**General Autonomic Function**	**Allgemeine Autonome Funktion**	**General Autonomic Function**	**Allgemeine Autonome Funktion**
**Subsection 1 'Autonomic control of the heart'**	**A "Autonome Kontrolle des Herzens"**	**Cardiovascular**	**Herzkreislauf**
At rest, do you have a heart rate lower than 60 beats per minute?<br><br>(if yes, check 'bradycardia')	Haben Sie einen Ruhepuls (Herzschlag in Ruhe) von weniger als 60 Schlägen pro Minute?<br><br>(wenn ja, wählen Sie "Bradykardie")	While measuring BP and pulse, the following question should also be asked at 1-min intervals:<br><br>Do you currently experience one or more of the following symptoms: Lightheadedness, dizziness, blurred vision, tingling in your ears, pounding/pulsing in your head goose bumps, nausea, or something else (note)	Während der Messung von BP und Puls sollte folgende Frage auch im 1-min-Intervall gestellt werden:<br><br>Empfinden Sie aktuell eine oder mehrere der folgenden Symptome: Benommenheit, Schwindel, Verschwommenes Sehen, Kribbeln in den Ohren, Hämmern/ Pulsieren im Kopf Gänsehaut, Übelkeit, oder etwas anderes (notieren)
At rest, do you have a heart rate greater than 100 beats per minute?<br><br>(if yes, check 'tachycardia')	Haben Sie einen Ruhepuls (Herzschlag in Ruhe) von mehr als 100 Schlägen pro Minute?<br><br>(wenn ja, wählen Sie "Tachykardie")	The patient should describe activities that may trigger the previously mentioned symptoms.	Der Patient sollte Aktivitäten beschreiben, die die zuvor genannten Symptome auslösen können.
Have you ever experienced, or been told that you have an arrhythmia, or irregular heart rate?<br><br>(If yes, check 'other dysrhythmias')	Haben Sie jemals erlebt, oder wurde Ihnen gesagt, dass Sie einen unregelmässigen Herzschlag oder eine Herzrhythmusstörung haben? (Wenn ja, wählen Sie "andere Rhythmusstörungen")	Are there measures/tactics how you can prevent or mitigate these symptoms?	Gibt es Maßnahmen/Taktiken, wie Sie diese Symptome verhindern oder mildern können?

(If 'I don't know' then check 'unknown'),<br><br>(If unable to assess, then check 'Unable to assess')<br>(If 'no' to all, then check 'normal)	(Wenn "ich weiss nicht", wählen Sie "unbekannt")<br>(Wenn nicht in der Lage zu beurteilen, wählen Sie "nicht in der Lage zu beurteilen")<br>(Wenn "nein" bei allen, wählen Sie "normal")		

**Subsection 2 'Autonomic control of blood pressure'**	**B "Autonome Kontrolle des Blutdrucks"**	(1)	(1)
At rest, do you have a systolic blood pressure lower than 90mmHg?<br><br>(If yes, check 'resting systolic blood pressure below 90mmHg')	Haben Sie in Ruhe einen systolischen Blutdruck tiefer als 90mmHg?<br><br>(wenn ja, wählen Sie "systolischer Blutdruck unter 90mmHg")	(1)	(1)
In the last 6 months, have you experienced, or been told that you have a condition called 'Orthostatic Hypotension'?<br><br>(if yes, check 'orthostatic hypotension')	Haben Sie in den letzten 6 Monaten eine "orthostatische Hypotonie" erlebt, oder wurde Ihnen gesagt, dass Sie eine "orthostatische Hypotonie" haben?<br><br>(wenn ja, wählen Sie "orthostatische Hypotonie")	(1)	(1)
Have you ever experienced, or been told that you have a condition called 'Autonomic Dysreflexia'?<br><br>(if yes, check 'Autonomic Dysreflexia')	Haben Sie jemals erlebt, oder wurde Ihnen gesagt, dass Sie eine "autonome Dysreflexie" haben?<br><br>(wenn ja, wählen Sie "autonome Dysreflexie")	(1)	(1)

(If 'I don't know' then check 'unknown')<br>(If unable to assess, then check 'Unable to assess')<br>(If 'no' to all, then check 'normal)	(Wenn "ich weiss nicht", wählen Sie "unbekannt")<br>(Wenn nicht in der Lage zu beurteilen, wählen Sie "nicht in der Lage zu beurteilen")<br>(Wenn "nein" bei allen, wählen Sie "normal")	(1)	(1)

**Subsection 3 'Autonomic control of sweating'**	**C "Autonome Kontrolle des Schwitzens"**	**Sudomotoric**	**Sudomotoric**
Do you sweat the same amount and as often now, compared to before your injury?<br><br>(if yes, then check 'normal' and no need to ask #2, #3, or #4)	Schwitzen Sie genauso viel und genauso oft wie vor Ihrer Verletzung?<br><br>(wenn ja, wählen Sie "normal" und #2, #3 und #4 müssen nicht gefragt werden)	Are you able to sweat during exercise or when exposed to hot environments?<br>(if no, then check 'Absent` and no need to ask #2, #3 and #4)	Schwitzen Sie beim Sport oder in heissen Umgebungen?<br><br>(wenn ja, wählen Sie "absent" und müssen nicht gefragt werden)
Compared to before your injury, do you sweat a greater amount above the level of your injury?<br><br>(if yes, check 'hyperhydrosis above lesion')	Im Vergleich zu vor Ihrer Verletzung, schwitzen Sie vermehrt im Bereich oberhalb Ihrer Verletzungshöhe?<br>(wenn ja, wählen Sie "Hyperhydrose oberhalb der Läsion")	Do you sweat above your Neurological Level of Injury the same amount, a greater amount, a lower amount or not at all (during exercise or when exposed to hot environments)?	Schwitzen Sie im Bereich oberhalb ihres neurologischen Verletzungsniveaus genauso viel, mehr, weniger oder gar nicht (beim Sport oder in heissen Umgebungen)?
Compared to before your injury, do you sweat a greater amount below the level of your injury?<br><br>(if yes, check 'hyperhydrosis below lesion')	Im Vergleich zu vor Ihrer Verletzung, schwitzen Sie vermehrt im Bereich unterhalb Ihrer Verletzungshöhe?<br>(wenn ja, wählen Sie "Hyperhydrose unterhalb der Läsion")	Do you sweat below your Neurological Level of Injury the same amount, a greater amount, a lower amount or not at all (during exercise or when exposed to hot environments)?	Schwitzen Sie im Bereich unterhalb ihres neurologischen Verletzungsniveaus genauso viel, mehr, weniger oder gar nicht (beim Sport oder in heissen Umgebungen)?

Compared to before your injury, do you sweat a lower amount below the level of your injury?<br><br>(if yes, check 'hypohydrosis below lesion')	Im Vergleich zu vor Ihrer Verletzung, schwitzen Sie weniger im Bereich unterhalb Ihrer Verletzungshöhe?<br>(wenn ja, wählen Sie "Hypohydrose unterhalb der Läsion")	Are there other circumstances or situations in which you sweat (e.g., if you have a full bladder or are sick)?	Gibt es andere Umstände oder Situationen in den sie schwitzen (z.B., wenn Sie eine volle Harnblase haben oder krank sind)?
(If 'I don't know' then check 'unknown')<br>(If unable to assess, then check 'Unable to assess')	(Wenn "ich weiss nicht", wählen Sie "unbekannt")<br>(Wenn nicht in der Lage zu beurteilen, wählen Sie "nicht in der Lage zu beurteilen")		

**Subsection 4 'Temperature Regulation'**	**D "Temperaturregulation"**	**Thermoregualation Core Body Temperature**<br>**(2) Replaced through data collection (Measuring Core Body Temp)**	**Thermoregulation, Körperkerntemperatur**<br>**(2) Ersatz durch Datenkollektion (Messung der Körperkerntemperatur)**
Has your ability to control your own body temperature changed since your injury?<br><br>(if 'no', check 'normal' and no need to ask #2 or #3)	Hat sich Ihre Fähigkeit Ihre Körpertemperatur zu regulieren sich verändert seit Ihrer Verletzung?<br><br>(wenn nein, wählen Sie "normal" und #2 oder #3 müssen nicht gefragt werden)	(2)	(2)
Do you have a difficult time remaining cool in a hot environment?<br><br>(if yes, then check 'hyperthermia')	Haben Sie Schwierigkeiten in einer heissen Umgebung kühl zu bleiben?<br><br>(wenn ja, wählen Sie "Hyperthermie")	(2)	(2)

Do you have a difficult time remaining warm in a cool environment?<br>(if yes, then check 'hypothermia')	Haben Sie Schwierigkeiten in einer kühlen Umgebung warm zu bleiben?<br><br>(wenn ja, wählen Sie "Hypothermie")	(2)	(2)
(If 'I don't know' then check 'unknown')<br>(If unable to assess, then check 'Unable to assess')	(Wenn "ich weiss nicht", wählen Sie "unbekannt")<br>(Wenn nicht in der Lage zu beurteilen, wählen Sie "nicht in der Lage zu beurteilen")	(2)	(2)

**Subsection 5 'Autonomic and somatic control of bronchopulmonary system'**	**E "Autonome und somatische Kontrolle des bronchopulmonalen Systems"**	**Bronchopulmonary System (Comparing with Data from Clinic Information System = Medfolio)<br>+ Spirometrie**	**Bronchopulmonales System (Vergleichen mit Informationen aus dem Klinik Information System = MedFolio)<br>+ Spirometrie**
Has your ability to control your breathing/coughing/respiration changed since your injury?<br><br>(if 'no change', check normal and no need to ask #2-4)	Hat sich die Fähigkeit, Ihr Atmen und Husten zu beeinflussen und zu kontrollieren seit Ihrer Verletzung verändert?<br>(wenn nein, wählen Sie "normal" und #2-4 müssen nicht gefragt werden)		
Are you unable to voluntarily breathe without ventilator support?<br><br>(if yes, check 'unable to breathe requiring full ventilator support')	Sind Sie auf ein Beatmungsgerät angewiesen?<br>(wenn ja, wählen Sie "Spontanatmung beeinträchtigt, benötigt komplette Beatmungsunterstützung")		

Do you have impaired voluntary breathing requiring ventilator support part of the time?<br><br>(if yes, check 'Impaired voluntary breathing requiring partial vent support')	Ist Ihre Atmung beeinträchtigt und brauchten Sie teilweise ein Beatmungsgerät?<br>(wenn ja, wählen Sie "Spontanatmung beeinträchtigt, benötigt teilweise Beatmungsunterstützung")		
Do you have impaired voluntary respiration, but do not require ventilator support at any time?<br>(if yes, 'Voluntary respiration impaired, does not require vent support')	Ist Ihre Atmung beeinträchtigt, aber Sie brauchten zu keiner Zeit ein Beatmungsgerät?<br>(wenn ja, wählen Sie "Spontanatmung beeinträchtigt, braucht keine Beatmungshilfe")		
(If 'I don't know' then check 'unknown')<br>(If unable to assess, then check 'Unable to assess')<br>(If 'no' to all, then check 'normal)	(Wenn "ich weiss nicht", wählen Sie "unbekannt")<br>(Wenn nicht in der Lage zu beurteilen, wählen Sie "nicht in der Lage zu beurteilen")		

**'Lower urinary tract, bowel and sexual function'**	**"Untere Harntrakt-, Darm- und Sexualfunktion"**		
**Bladder**<br>Do you have an awareness of the need to empty your bladder?<br><br>Do you have the ability to prevent leakage (continence)?<br><br>What is your usual method for emptying the bladder?	**Harnblase**<br>Nehmen Sie wahr, wenn Sie Ihre Harnblase entleeren müssen?<br><br>Haben Sie die Fähigkeit Urinverlust (Kontinenz) zu vermeiden?<br><br>Was ist Ihre übliche Methode, Ihre Harnblase zu entleeren?	**Bladder**<br><br>**Awareness bladder fullness**<br>Do you have lower abdominal sensation of bladder filling?<br><br>**Ability to prevent bladder leakage**<br>Do you have lower abdominal sensation of bladder filling?	**Harnblase**<br><br>**Bewusstsein für die Notwendigkeit der Harnblasenentleerung:**<br>Spüren Sie die Füllung der Harnblase im Bereich des Unterbauches?<br><br>**Urinkontinenz:**<br>Spüren Sie die Füllung der Harnblase im Bereich des Unterbauches?

**Bowel**<br>Do you have the sensation of need for a bowel movement?<br><br>Do you have the ability to prevent stool leakage (continence)?<br><br>Do you have voluntary anal sphincter contraction?	**Darm**<br>Spüren Sie, wenn Sie Ihren Darm entleeren müssen?<br><br>Haben Sie die Fähigkeit Stuhlverlust (Kontinenz) zu vermeiden?<br><br>Können Sie willentlich den Schliessmuskel des Enddarms (Analsphinkter) zusammenziehen?	**Bowel**<br>**Awareness of bowel fullness**<br>*Do you have sensation in your abdominal and/or pelvic area of the need to move your bowels?*<br><br>**Ability to prevent bowel leakage**<br>*Can you hold your bowels if you feel the need to have a bowel movement?*	**Darm**<br>**Notwendigkeit zur Entleerung des Darms:**<br>Spüren Sie im Bauch- oder Beckenregion, wenn Sie ihren Darm entleeren müssen?<br><br>**Stuhlkontinenz:**<br>Können Sie ihren Stuhlgang zurückhalten, wenn Sie merken, dass Sie ihren Darm entleeren müssten?

**Sexual Function**	**Sexualfunktion**	**Genitalia and Reproductive Organs Function**	**Genitalien und Funktion der Fortpflanzungsorgane**
Are you able to experience genital arousal (erection/vaginal lubrication/wetness)<br><br>By thinking sexual thoughts? By reflex or touch?<br><br>Are you able to experience orgasm?<br><br>Are you able to ejaculate? (M only)<br><br>Do you have the sensation of menses? (F only)	Können Sie Erregungen im Genitalbereich erleben (Gliedversteifung/ vaginale Sekretion)<br><br>Durch sexuelle Fantasien? Als Reflex oder durch Berührung? Erleben Sie einen Orgasmus?<br><br>Können Sie ejakulieren (Samenerguss)? (nur Männer)<br><br>Spüren Sie Ihre Menstruation? (nur Frauen)	**Do you have genital arousal, e.g., erections or lubrication when are psychologically sexually aroused?**<br>- if yes, just as you did prior to the injury or different?<br><br>**Do you have genital arousal, i.e., erections or lubrication, when your genitals are touched?**<br>- if yes, just as you did prior to the injury or different?<br><br>**Are you able to achieve orgasm with sexual stimulation?**<br>- if yes, just as you did prior to the injury or different?	**Verspüren Sie genitale Erregung, z. B. Erektionen oder Lubrikation, wenn Sie psychisch sexuell erregt sind?**<br>- Wenn ja, genauso wie vor der Verletzung oder anders?<br><br>**Verspüren Sie genitale Erregung, d. h. Erektionen oder Lubrikation, wenn Ihre Genitalien berührt werden?**<br>- Wenn ja, genauso wie vor der Verletzung oder anders?<br><br>**Sind Sie in der Lage, bei sexueller Stimulation einen Orgasmus zu erreichen?**<br>- Wenn ja, genauso wie vor der Verletzung oder anders?

		**Are you able to achieve ejaculation with sexual stimulation?**<br>- if yes, just as you did prior to the injury or different?	**Sind Sie in der Lage, bei sexueller Stimulation eine Ejakulation zu erreichen?**<br>- Wenn ja, genauso wie vor der Verletzung oder anders?
Analysis:<br><br>Normal function = 2<br>Reduced or altered neurologic function = 1<br>Complete loss of control = 0 Unable to assess due to preexisting or concomitant problems<br>= NT	Analyse:<br><br>Normale Funktion = 2<br>Reduzierte oder veränderte neurologische Funktion = 1<br>Komplett fehlende Funktion = 0<br>Aufgrund von vorbestehenden oder begleitenden Problemen nicht beurteilbar = NT		

Detailed Assessment: Adaptations were mainly justified to save time and organization issues. The idea was that a physician should be able to perform the examination on the ward in a low-threshold manner. The examination should not take too long and should be feasible with simple equipment to make this possible. In addition, there is often no routine support person available in the daily routine of the ward. Therefore, it should be possible for the assessment to be carried out by a single person. We are aware that the adjustments could deteriorate the quality of examination quality and measured data. The aim should be to make the examination as simple as possible but to influence the quality of the measurements as little as possible. In the future, further research is certainly necessary to analyze these points and optimize the assessment. For the questionnaire may be predefined examples could be helpful for a better understanding for the participants.

Measurements: First, systolic and diastolic blood pressure [mmHg] and pulse [bpm] were measured in a supine position. At least five measurements were taken at intervals of one minute. If no significant changes occurred after the first five minutes, no further measurements were performed at the examiner's discretion. If substantial changes occurred, a total of ten measurements were taken. After completing the measurements in the supine position, the participant was asked once whether he experienced symptoms of low blood pressure during the measurement period. After that, the participant was transferred to the sitting position. Sitting was defined as raising the headrest to a sitting position of the patient (no measurement/minimal angle was defined) or sitting freely on the edge of the bed with the legs flexed about 90° and the feet on the floor. Blood pressure and pulse were again measured in the supine position (five or ten times in a row) in the sitting position. In a sitting position, we asked every minute whether the patient felt symptoms of low blood pressure. The blood pressure was measured with the Nellcor patient monitor N5600 with a cuff placed on the left upper arm. For the heart rate, we used the Nellcor patient monitor N5600 with three ECG electrodes placed on the upper body. The body temperature, in centigrade Celsius [C°], was measured orally with a clinical thermometer in a sublingual position simultaneously to blood pressure measurements. At least two measurements were conducted. If the measured values did not differentiate more than 0.2 C°, then no third measurement was made. If the difference was greater than 0.2 C°, a third measurement was conducted. When using alternative methods for measuring body temperature, such as oral or axillary positioning, it was noted accordingly. Spirometry was performed only through assessor 1 and could only be done when the spirometer was available. The measurements were taken in an upright position (elevated headrest or sitting freely at the edge of the bed) with a nose clip applied. We used a Micro Medical ML3535 MK8 MicroLoop von Care Fusion hand spirometer, which was calibrated for every new examination. In each case, three measurements were taken, with the best measurement being used for the score. We documented the forced vital capacity (FVC) [ml], bodyweight and FVC per bodyweight [ml/kg]. The participant's body weight was obtained through medical charts or from the participant. A neurological examination is needed for the sacral autonomic part of the assessment. We tested sensitivity from T11-L2 and S3-5 in accordance with the International Standards for Neurological Classification of SCI. Twisted cotton swabs and safety pins (size 50mm) were used. Further, we tested the deep anal pressure and voluntary anal contraction (scored as normal, abnormal, absent, not tested or not testable) as well as the anal reflex and/or the bulbocavernosus reflex (scored as normal, increased, and decreased, absent, not tested, not testable). Comments could be noted in unique columns.
