# Supplementary material for: German Transcreation of the International Standards to Document Remaining Autonomic Function After Spinal Cord Injury (Second Edition): A Feasibility Study in Individuals With Subacute Phase Spinal Cord Injury/Disease (SCI/D)
Source: Top Spinal Cord Inj Rehabil. 2025 Aug 22;31(3):89–100. doi: 10.46292/sci25-00010 (PMC12376144; doi:10.46292/sci25-00010)
Supplement: Supplementary file 2 [file i1945-5763-31-3-89_s02.pdf]

SUPPLEMENTARY RESULTS

Full list, key points of feedback round and additional observations

Questionnaire:

- Sudomotor function: Across all 3 assessments, the first question was answered 4 times with no = "anhidrosis." ISAFSCI protocol suggests no further questions should be asked. Since we decided to ask all questions in sudomotor function, we saw in 2 of the 4 assessments participants stated to be able to sweat.
- In general, problems were expressed in answering the questions adequately, as there was no opportunity to deal with the topics as part of the initial rehabilitation. This applies to the topics of sexual function and sudomotor function.

Measurements:

- Body temperature: In 3 assessments, the body temperature was measured 3 times, even though only 2 measurements were necessary.
- Spirometry: Bedside spirometry was found to be quite complex and time-consuming.

Scoring sheet and assessment:

- Pulse measurement: For scoring, it was unclear how to handle the measured data to evaluate the score (taking average values, consider the range [min/max]).
- Sacral autonomic function: Some combinations of assessment results lead to an unclear score, for example, deciding between "altered" or "absent" scores for ejaculation when both normal sensation of T11-12 and absent sacral reflexes are present.
- Timing: The term "timing" in sacral autonomic function remained unclear and may be omitted in future assessments.

eTable 1. Questionnaire and ISAFSCI score-sudomotor function

ID	Assessment	Question 1: Ability to sweat in hot environment or exercise	Question 2: Ability to sweat above NLI	Question 3: Ability to sweat below NLI	Question 4: Ability to sweat in other circumstances	ISAFSCI Score
1	1	yes	absent	absent	absent	2
2	1	yes	more	less	equal	1
3	1	yes	equal	equal	equal	2
4	1	yes	equal	less	absent	1
5	1	no	absent	absent	less	0
6	1	yes	less	absent	absent	0
7	1	yes	equal	equal	unknown	0
8	1	yes	equal	equal	absent	2

(continues)

This supplementary digital material accompanies
Top Spinal Cord Inj Rehabil 2025;31(3):89-100
© 2025 American Spinal Injury Association
www.asia-spinalinjury.org
doi: 10.46292/sci25-00010

eTable 1. Questionnaire and ISAFSCI score-sudomotor function *(cont.)*

ID	Assessment	Question 1: Ability to sweat in hot environment or exercise	Question 2: Ability to sweat above NLI	Question 3: Ability to sweat below NLI	Question 4: Ability to sweat in other circumstances	ISAFSCI Score
9	1	unknown	unknown	unknown	absent	9
10	1	yes	equal	absent	absent	0
11	1	yes	equal	absent	more	0
12	1	yes	equal	more	absent	2
1	2	yes	equal	equal	absent	2
2	2	yes	equal	less	absent	1
3	2	yes	equal	equal	absent	2
4	2	yes	equal	less	absent	1
5	2	no	absent	absent	less	0
6	2	yes	equal	absent	equal	0
7	2	yes	more	absent	absent	0
8	2	yes	equal	equal	absent	2
9	2	no	Not tested	Not tested	absent	0
10	2	yes	equal	absent	absent	0
11	2	yes	more	absent	absent	0
12	2	yes	equal	equal	absent	2
1	3	yes	equal	equal	absent	1
2	3	yes	equal	absent	absent	1
3	3	yes	equal	equal	absent	2
4	3	yes	equal	less	absent	1
5	3	no	less	absent	more	0
6	3	yes	equal	absent	equal	0
7	3	yes	more	absent	absent	2
8	3	yes	more	absent	equal	0
9	3	unknown	unknown	equal	unknown	9
10	3	yes	less	absent	absent	0
11	3	yes	more	absent	absent	0
12	3	yes	equal	equal	absent	1

eTable 2. Questionnaire and ISAFSCI score – thermoregulation core body temperature

ID	Assessment	Temperature measurement 1 [°C]	Temperature measurement 2 [°C]	Delta [°C]	Delta >0.2°C	Temperature measurement 3 [°C]	Type
1	1	34.9	35.2	0.3	yes	Not Tested	1,2
2	1	36.3	36.4	0.1	no		2,3
3	1	35.8	36.0	0.2	no	36.0	2
4	1	36.1	36.2	0.1	no		2
5	1	35.2	35.4	0.2	no		2
6	1	37.0	37.0	0	no		3
7	1	35.7	36.0	0.3	yes	36.0	2
8	1	36.6	36.0	0.6	yes	36.8	2,3
9	1	36.5	36.5	0	no		3
10	1	36.5	36.4	0.1	no		3
11	1	36.6	36.6	0	no		3
12	1	35.5	35.6	0.1	no	35.9	2
1	2	35.2	35.1	0.1	no		2
2	2	36.1	36.1	0	no		2
3	2	36.4	36.5	0.1	no		3
4	2	36.2	35.4	0.8	yes	35.6	2
5	2	36.4	36.5	0.1	no		3
6	2	37.0	37.1	0.1	no		3
7	2	35.8	35.8	0	no		2
8	2	36.4	36.5	0.1	no		3
9	2	36.4	36.4	0	no		3
10	2	36.5	36.5	0	no		3
11	2	36.6	36.7	0.1	no		3
12	2	36.5	36.5	0	no		3
1	3	36.5	36.5	0	no		3
2	3	35.5	36.2	0.7	yes	35.7	2
3	3	36.4	36.6	0.2	no		3
4	3	36.2	36.2	0	no		2
5	3	35.8	36.2	0.4	yes	36.2	2
6	3	36.4	36.4	0	no		3
7	3	36.1	35.6	0.5	yes	36.1	2
8	3	35.6	36.4	0.8	yes	36.5	2

(continues)

eTable 2. Questionnaire and ISAFSCI score – thermoregulation core body temperature *(cont.)*

ID	Assessment	Temperature measurement 1 [°C]	Temperature measurement 2 [°C]	Delta [°C]	Delta >0.2°C	Temperature measurement 3 [°C]	Type
9	3	36.3	34.6	1.7	yes	34.5	1
10	3	35.4	35.5	0.1	no		2
11	3	36.4	36.4	0	no	36.5	3
12	3	36.5	36.6	0.1	no		3

Note: Type of temperature condition: 1 = hypothermia ≤35 °C; 2 = subnormal 35.1-36.3 °C; 3 = normal 36.4-37.6 °C; 4 = elevated 37.7-37.9 °C; 5 = hyperthermia ≥38.0 °C.

eTable 3. Number of potentially influencing factors, total per item, preparation questions

**Consumption/activities of potentially influencing factors four hours before assessment**		
**Factors**	**Applies [*n*]**	**Does not apply [n]**
Caffeine	21	15
Big meal	16	20
Heavy training	10	26
Nicotine	9	27
Cannabis	0	36
Alcoholic beverages	0	36

eTable 4. Number of potentially influencing factor per assessment per person across all three assessments

**Applying number of potentially influencing factors per assessment per person**		
	n	**%**
None	6	17
1 of 6	12	33
2 of 6	10	28
3 of 6	8	22
>3 of 6	0	0

eTable 5. Number participants wearing compression garments across all three assessments

**Wearing of compression garments**				
	**Compression stockings**		**Abdominal belt**	
	n	**%**	***n***	**%**
Yes	23	64	5	14
No	13	36	30	83
Unknown	0	0	0	0
Missing information	0	0	1	3

eTable 6. Time since last bladder emptying across all three assessments

**Time since the last bladder emptying**	
	**min**
Median	120
Lower quartile	60
Upper quartile	150
Minimum	0
Maximum	300

eTable 7. Number of participants - bladder emptying across all three assessments

**Information about bladder emptying**		
**Is the bladder emptied?**	***n***	**%**
Yes	22	61
No	10	28
Not tested	4	11

eTable 8. Number of participants - bowel emptying across all three assessments

**Information about bowel emptying**		
**Last bowel movement**	***n***	**%**
Today	28	78
1 days ago	6	17
2 days ago	2	5

eTable 9. Number of participants - bladder and bowel emptying combined across all three assessments

**Combined bladder and bowel emptying**		
	**n**	**%**
Both empty	15	42
One of both	17	47
None	1	3
Not tested	0	0
Missing information	3	8
